# Identification of regulatory networks involved in microglia subset specification

**DOI:** 10.1002/alz70855_106777

**Published:** 2025-12-24

**Authors:** Jason Ngo, Emily Lee, Nivedita Nimmagadda, Vilas Menon, Falak Sher, Marta Olah

**Affiliations:** ^1^ Columbia University Medical Center, New York, NY, USA; ^2^ Taub Institute for Research on Alzheimer's Disease and the Aging Brain, New York, NY, USA; ^3^ Center for Translational and Computational Neuroimmunology, New York, NY, USA; ^4^ Center for Translational & Computational Neuroimmunology, Columbia University Irving Medical Center, New York, NY, USA

## Abstract

**Background:**

We have recently established that microglia in the aged and AD human brain exist in discrete, transcriptionally defined states that have complex associations with clinicopathological features of AD (PMID: 33257666). This multifaceted relationship made it clear that we should develop approaches that allow for fine‐tuning of microglia population structure in order to achieve therapeutic benefit in AD. However, how Ad genes affect the cell intrinsic regulatory networks that shape human microglia phenotypes is yet unknown. To fill in this gap we investigated how AD genes affect the transcriptional regulatory networks that underlie microglia phenotypic specification.

**Method:**

To achieve this goal, we utilized CROP‐seq, that enables pooled CRISPR screens with single‐cell transcriptome resolution. To assemble the guide RNA (gRNA) library for this study, we focused on nascent AD drug targets that were nominated by the National Institute on Aging's AMP‐AD consortium and TREAT‐AD centers and were expressed in microglia. gRNA library transduction was performed according to our recently published protocol (PMID: 38600587), and the cells were then submitted to a modified single cell RNA‐sequencing pipeline on the 10x Genomics Chromium platform.

**Result:**

Generally, we found that genetic perturbation of AD genes in microglia resulted in changes in transcriptional regulatory networks associated with inflammation and lipid metabolism. More specifically, interestingly, the microglia subsets that were most affected by perturbations in AD genes were the previously described cluster 8 and cluster 9 microglia (PMID: 33257666). Previously we found cluster 8 microglia subset to be enriched in genes associated with metabolic changes while cluster 9 was characterized by genes involved cell proliferation. Both of these subsets had a negative association tau pathology in a large ROS/MAP transcriptomic dataset, suggesting that these microglia phenotypes might be protective in AD and that perturbations in AD genes might dysregulate the transcriptional regulatory networks involved in phenotypic specification of these subsets.

**Conclusion:**

Our findings prioritize two human microglia subsets as being primarily affected by perturbations in AD genes, and identify transcriptional regulatory networks that could be targeted to modulate microglia population structure in a way that will potentially support the maintenance and/or restoration of tissue homeostasis in AD.

